# The Influence of Community Health Resources on Effectiveness and Sustainability of Community and Lay Health Worker Programs in Lower-Income Countries: A Systematic Review

**DOI:** 10.1371/journal.pone.0170217

**Published:** 2017-01-17

**Authors:** Daniel H. de Vries, Robert Pool

**Affiliations:** Department of Anthropology, University of Amsterdam, Amsterdam, The Netherlands; The Chinese University of Hong Kong, HONG KONG

## Abstract

**Background:**

Despite the availability of practical knowledge and effective interventions required to reduce priority health problems in low-income countries, poor and vulnerable populations are often not reached. One possible solution to this problem is the use of Community or Lay Health Workers (CLHWs). So far, however, the development of sustainability in CLHW programs has failed and high attrition rates continue to pose a challenge. We propose that the roles and interests which support community health work should emerge directly from the way in which health is organized at community level. This review explores the evidence available to assess if increased levels of integration of community health resources in CLHW programs indeed lead to higher program effectiveness and sustainability.

**Methods and Findings:**

This review includes peer-reviewed articles which meet three eligibility criteria: 1) specific focus on CLHWs or equivalent; 2) randomized, quasi-randomized, before/after methodology or substantial descriptive assessment; and 3) description of a community or peer intervention health program located in a low- or middle-income country. Literature searches using various article databases led to 2930 hits, of which 359 articles were classified. Of these, 32 articles were chosen for extensive review, complemented by analysis of the results of 15 other review studies. Analysis was conducted using an excel based data extraction form. Because results showed that no quantitative data was published, a descriptive synthesis was conducted. The review protocol was not proactively registered. Findings show minimal inclusion of even basic community level indicators, such as the degree to which the program is a community initiative, community input in the program or training, the background and history of CLHW recruits, and the role of the community in motivation and retention. Results show that of the 32 studies, only one includes one statistical measure of community integration. As a result of this lack of data we are unable to derive an evidence-based conclusion to our propositions. Instead, our results indicate a larger problem, namely the complete *absence* of indicators measuring community relationships with the programs studied. Studies pay attention only to gender and peer roles, along with limited demographic information about the recruits. The historicity of the health worker and the community s/he belongs to is absent in most studies reviewed. None of the studies discuss or test for the possibility that motivation emanates from the community. Only a few studies situate attrition and retention as an issue enabled by the community. The results were limited by a focus on low-income countries and English, peer-reviewed published articles only.

**Conclusion:**

Published, peer-reviewed studies evaluating the effectiveness and sustainability of CLHW interventions in health programs have not yet adequately tested for the potential of utilizing existing community health roles or social networks for the development of effective and sustainable (retentive) CLHW programs. Community relationships are generally seen as a “black box” represented by an interchangeable CLHW labor unit. This disconnect from community relationships and resources may have led to a systematic and chronic undervaluing of community agency in explanations of programmatic effectiveness and sustainability.

## Introduction

The coincidence of the halfway mark to the millennium development goals (MDGs) with the 30th anniversary of Alma-Ata stimulated discussion about the role of revitalized primary health care in the strengthening of health systems in low- or middle-income countries [1,2]. One of the lasting impressions of these discussions is the difficulty of motivating community ownership and participation in health, including the successful expansion of community health workers. Explicitly addressed as one of Alma-Ata’s principles, the ability of poor communities to participate in health service delivery appears to have been one of the least fulfilled elements of the Alma-Ata philosophy. The effectiveness of community health worker programs has been considered “patchy”, with difficulties in scale-up, an observed lack of consistent supervision, weak linkages to existing health systems, and no sustained community financing [3–6]. Unfortunately, in the new United Nations Sustainable Development Goals, community participation does not surface as a central theme in any of the formulations, with the exception of the goal to ensure availability and sustainable management of water and sanitation (Goal 6./b) [7]. Yet, the increasing awareness of a global shortage of human resources for health, particularly in low- and middle-income countries [8,9], as well as the observed inequity in health systems [10,11], emphasizes the continued need to strengthen linkages to the community and to reinstate community health workers [1,2].

To achieve effective and sustainable community participation, we propose that health service delivery systems should emerge from the way in which health is organized at community level. Our hypothesis is that inclusion into programmatic design of local structures, networks and roles which do not necessarily have an explicit medical function increases the effectiveness and sustainability of community and lay health worker programs. The aim of this systematic review, therefore, is to assess what empirical evidence exists that may confirm this proposition. We define community health workers as a broad category of lay workers identified as being able to carry out functions related to health care delivery at community level without a formal professional or paraprofessional certificate or tertiary education degree. In the literature and in practice, various terms have been used for this rising health cadre, most commonly and historically “community health worker”, but also “peer health worker”, “non-professional health care worker” and “lay health worker”. In this paper we will refer to this cadre as Community and Lay Health Workers (CLHW).

The lay worker concept in health service delivery received much initial enthusiasm in the 1980s, but waned as scaling-up of local CLHW program models appeared difficult [3]. One major issue plaguing community and lay health worker programs is high levels of attrition resulting from resignations, terminations, or relocations [2,4,12], leaving the few enthusiastic and reliable lay workers that remain to become overloaded with work [13–15]. Attrition rates have been reported of up to 30% over 9 months in Senegal and 50% over 2 years in Nigeria [16], while Olang'o et al. report an attrition rate of 33% among home-based care community health workers in western Kenya [17]. Furthermore, although community-based lay health workers can be volunteers [18], in practice most are financially rewarded, while there are hardly any examples of sustained community financing in low- or middle-income contexts [19]. High attrition rates reduce the stability of programs, increase training costs because of the need for continuous replacement, and make programs difficult to manage [20–22]. Moreover, fee-for-service payments may encourage inappropriate treatment [2].

Despite the significant impact of community and lay health worker attrition on programmatic stability and effectiveness, a World Health Bulletin points to the paucity of data on this issue [23]. These authors argue that lay worker attrition has been neither a measurement priority nor a research priority. We believe that this conclusion is remarkable, if attrition is seen as a factor leading to a lack of continuity in the relationship between CLHWs and their community. The question then emerges: to what extent is the quality of the relationship between the community and the CLHW essential for the effective and sustainable working of community health worker programs in low- or middle-income countries? An initial reading of the literature suggests that the community relationship is indeed of significant importance. In a global review, UNICEF notes that effective CLHW programs are partly dependent on frequent interactions with community members [19]. While it is noted that supervision is often one of the weakest links in many CLHW programs, it has also been found that it is mostly effective at small scales only because a significant amount of supervision and oversight comes from the community itself [24]. There is evidence suggesting that acceptance, support, and respect from the community as well as from the formal health system is essential for the motivation and effectiveness of CLHWs, and that CLHWs should be selected on the basis of their motivation to serve the community in which they work [25]. Belonging to the community is crucial because ultimately, the success of the CLHW program is measured at community level [26,27]. Landon qualitatively described an Alaskan CLHW program where “high retention communities” received more emergency, financial and material support and respect from the community along with greater responsiveness from village councils [21]. Others note that wherever selection of a CLHW has not been carefully considered, this can lead to a lack of trust from the community and become a contributing factor to high turnover [17,26,28]. Also noted is the need to pay attention to the economic and cultural environment within which CLHWs operate [17], such as gender norms [29].

These findings lead us to question whether the extent to which CLHWs are integrated within pre-existing community structures makes a difference with respect to sustainability (including attrition) and program effectiveness. Are CLHW programs which are inclusive and sensitive to indigenous community roles, groups and networks during program design phases intrinsically more effective and sustainable than those built on new roles and groups developed and implemented from the outside? We seek to identify peer-reviewed studies that evaluate CLHW programs in low- or middle-income settings using either a randomized, quasi-randomized or before/after evaluative methodology. The population of interest in this study is the CLHW participating in health programs in low- or middle-income settings. Our assumption here is that CLHW patterns in these settings differ substantially from patterns in high-income countries, as the latter are often destination sites for many health worker as a result of much more viable financial remuneration schemes. The intervention pertains to the influence of indicators of community integration in programmatic outcomes. Based on fieldwork experience, through a study on community health resources in Uganda (CoHeRe), we chose to pay specific attention to four potential indicators of community integration: 1) community-based planning of the CLHW program, 2) the history of CLHW recruits, 3) community input in training of CLHWs, and 4) community engagement during implementation of the CLHW program. The outcomes of interest are the observed effectiveness and general sustainability (the latter including CLHW retention) of the health programs studied.

## Method

### Definition of community and lay health workers

We view a CLHW program as a health program which has a strong relational component to the community through the inclusion of CLHWs who operate ultimately as liaisons between the health system and the community. We define CLHW as an umbrella term used for a heterogeneous group of lay people trained to promote health among their peers in communities [30]. CLHWs provide curative and promotional health services, mediate between communities and providers, and encourage discussion on health issues [31]. They include providers involved in both paid and voluntary care. Compared to the terms “lay providers” or “non-professional health care workers”, the term CHW better illustrates the continuous history of this cadre since the 1980s, including the recent literature on task shifting and human resources for health [24]. Further, the term also explicitly connects the community level [32]. The “lay health worker” label refers more to roles assumed by lay people trained to assist health professionals and to take over certain tasks from them [4,33]. These types of lay worker may work more remotely from the community. At the same time, because we want to know the extent to which community integration matters, we selected a broad range of lay health persons, with the aim of distinguishing levels of community integration irrespective of the labeling attributed by authors. Whether CHWs are part of the community or not, whether they live in the village or the neighborhood, is not a criterion for selection; we remained interested in those health workers who were usually part-time, either as volunteers or on a low salary; they are generally not civil servants or professional employees [32]. We define community here as the social network of relationships which has organized itself at the end of the health service delivery chain. As such, we do not view community as a strictly spatial concept. Nor do we adhere to the view that community is necessarily a cooperative and collaborative network, as we know that communities are dynamic and increasingly fluid forms of social organization, and typically not homogeneous [34].

### Review protocol

A review protocol S3 File was established by the authors at the beginning of the review but not registered prospectively as the review had already progressed too far at time of registration, but has been made available as part of the review process. The literature search was conducted in two phases. A first search without from database inception until mid-2012 was performed using the Cochrane Library review abstracts, Academic search premier, Web of science, Science Direct, Google Scholar, PubMed, and Annual Reviews. A number of journals was hand-searched, in particular the Journal of Human Resources for Health, and references of particularly relevant full-text articles were also searched. A second update search was conducted in February 2^nd^ 2015, starting in 2012, using Medline, Web of Science, Cochrane Library and Sociological Abstracts. Search strategies were translated for use on different search platforms/databases. S1 File shows an example of the search strategy used for the second search (2012–2015), which mimics the strategy of the first, more general search (inception– 2012). For both searches, the following English search term was used: community health workers/ OR nurses' aides/ OR (((allied health* OR community health* OR community based health* OR health extension OR kinship OR lay health* OR lay nurse OR peer health* OR non-specialist health* OR village health* OR village malaria) ADJ2 (worker* OR activist* OR personnel* OR volunteer* OR aide*)) OR natural helper* OR barefoot doctor*). This search was reduced by a selection of low- and middle-income countries derived from the Worldwide Governance Indicators (WGI) database and undoubled. Studies identified were included in a RefWorks bibliographic database and organized according to the extent to which they fitted the categories of effectiveness studies, review articles, and other contextual or qualitative narratives. The software was used to identify duplicates.

The following criteria were applied to identify studies for review:

1. The paper evaluates the effectiveness of CLHWs in a health program
2. The paper is published in a peer-reviewed journal
3. The paper uses a randomized, quasi-randomized clinical trial or before/after methodology to test or evaluate the effectiveness of CLHW programs; or alternatively has a substantial qualitative component supporting a descriptive assessment
4. The paper studies a CLHW program located in a low- or middle-income country or region within a country. We excluded high-income countries because of the higher likelihood that CLHWs are financially remunerated and work in an environment with many more public extension services, making lessons learned less comparable.

Two reviewers independently conducted a review of titles and abstracts and selected articles for review based on inclusion/exclusion criteria. Disagreements were resolved, after which full text of potentially relevant papers was retrieved. After analysis of this subset of full papers, the first author proceeded with the final selection of articles for inclusion in the review and coding, in consultation with the second author. Both authors agreed upon the data extraction form S2 File, which was created in Excel and used to integrate information. The datasheet was piloted on a number of papers not included, including review papers. For each study selected for inclusion, a data extraction form was used to collect data for analysis. The datasheet (see Appendix II) included general information about the paper (e.g. journal, author, type of data, length of study, etc.) as well as information regarding the questions indicating levels of integration of community dynamics into the CLHW program studied):

- *Community-based program planning*: To what extent do the reviewed programs build upon pre-existing indigenous networks and social roles? Who took the initiative for the CLHW program? And how much community input was there in the planning of the program before its implementation?
- *Community history of recruits*: To what extent were the recruited CLHWs already engaged in community health roles? Were they recruited to work in traditional or indigenous roles that have historical ties to the community, or were these new CLHW roles? What social position did they have in the community?
- *Community input in training*: After recruitment, to what extent did community members have input in the training curriculum, and was the training based on experience inside relevant communities?
- *Community engagement during implementation*: To what extent did CLHW programs forge ongoing relationships and connections to community processes and dynamics to sustain motivation during implementation (e.g. community based recruitment and supervision)?

We used a critical appraisal process that involves (i) filtering against minimum criteria, involving adequacy of reporting detail on the data sampling, collection and analysis, (ii) technical rigor of the study elements indicating methodological soundness and (iii) paradigmatic sufficiency, referring to researchers’ responsiveness to data and theoretical consistency [35]. Each of the studies was reviewed by the first author for these qualities and a review sheet was created in Excel. We concluded the critical appraisal with a table noting internal validity (score 1 = low and 5 = high) with regard to credibility (internal validity), transferability (external validity), dependability (reliability) and confirmability (objectivity) [36].

### Analysis

After examining a sizable number of peer-reviewed articles, we learned that little quantitative data has been published detailing our research question. This is surprising, given the body of qualitative data suggesting the importance of community relationships. As a result, no quantitative meta-analysis of the contribution of community indicators to programmatic sustainability and effectiveness was feasible. Instead, while remaining systematic in approach, the review strategy shifted to a descriptive synthesis. For each article, an assessment was made of the extent to which the article included quantitative or qualitative data on each of the sub-questions assessing levels of community integration, followed by an assessment of the extent to which these studies provide empirical evidence that community integration influences effectiveness, levels of attrition, and general sustainability of the CLHW program in question. A generally thematic approach was adopted which allowed for the generation of themes emerging from the studies along the *a priori* dimensions of participation identified above. Results were organized following these *a priori* themes as well as sub-themes which emerged.

## Results

### Data reviewed

**Fig 1:**
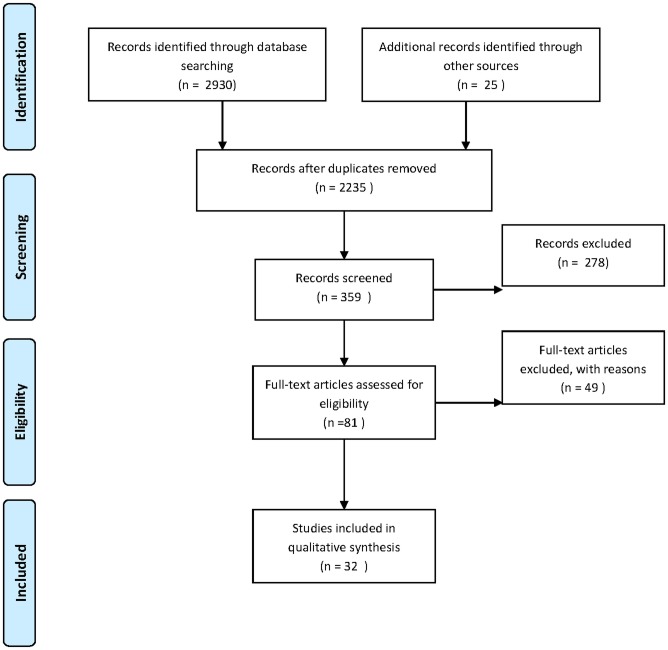
PRISMA flow diagram.

Fig 1 shows a PRISMA-based flow diagram of the articles included in this review. In total, our search produced 2235 unduplicated hits, out of which 359 articles were classified, based on their abstracts, as dealing with community health workers with regard to their relevance for the topic. Of these, 283 did not explicitly evaluate the effectiveness or retention of community health workers in programs, were not retraceable or were review papers themselves. After full reading, of the remaining articles, 49 studies were further excluded for review because they had not been conducted in a low- or middle-income country (these were pre-dominantly U.S.-based articles) or did not match the other criteria, leaving 32 articles for extensive review. Table 1 provides a summary of the final list of articles selected for review, including the country of the CLHW program (restricted to low- or middle-income countries), the publication type and the type of evidence. In addition, Table 1 provides the results of the bias of selected publications, which show some variability in the risk of bias, but score generally high.

**Table 1:** Summary of results and quality assessment.

Reference	Country	Publication type	Type of evidence	Credibility	Transferability	Dependability	Confirmability	Publication Bias Score
Adam et al. 2014	Kenya	PLOS One	Pre- and post test of 18 month to 3 year intervention	3	4	3	3	3
Agboatwalla & Akram 1995	Pakistan	Community Development Journal	Pre-post survey of 150 households with a 150 hh. control group of one year intervention	2	4	2	2	3
Baqui et al. 2009	Bangladesh	Journal of Tropical Medicine and International Health	Community-based cluster randomized controlled trial	5	5	5	5	5
Chang et al. 2009	Uganda	Journal of Acquired Immune Deficiency Syndromes	Retrospective cohort study over nine months	5	4	5	5	5
Chatterjee et al. 2014	India	Lancet	Randomised controlled trial at three sites	5	5	5	4	5
Colvin et al. 2003	South Africa	International Journal of Tuberculosis and Lung Disease	Non-randomized comparative study over two years	5	5	5	5	5
Debpuur et al. 2002	Ghana	Studies in Family Planning	Prospective cohort study over six years	5	4	5	5	5
Dick et al. 1996	South Africa	Tubercle and Lung Disease	Cohort study over six months supported by focus groups	5	4	5	5	5
Douthwaite & Ward 2005	Pakistan	Health Policy & Planning	Cross-sectional study of CLHWs working for at least four years	3	3	3	3	4
Dudley et al. 2003	South Africa	International Journal of Tuberculosis and Lung Disease	Non-randomised comparative study	5	5	5	5	5
Frazão & Marques 2009	Brazil	Rev Saúde Pública	Pre- and post test of one year intervention	5	4	5	5	5
Gazi et al. 2005	Bangladesh	Journal of Health and Population Nutrition	Pre- and post test of one year intervention with control and qualitative interviews	5	4	5	5	5
Hadi 2003	Bangladesh	Bulletin of the World Health Organization	Prospective cohort study of performance over three months	5	4	5	5	5
Harvey et al. 2008	Zambia	Malaria Journal	Non-randomized comparative study over one month	5	4	5	5	5
Jackson et al. 2013	South Africa	Journal of the International AIDS Society	Community cluster randomized controlled trial	5	5	5	5	5
Jacob et al. 2007	India	Acta Psychiatrica Scandinavia	Observation and review of performance	4	3	4	4	4
Jafar et al. 2010	Pakistan	BMJ	Cluster randomized controlled trial	5	5	5	5	5
Jennings et al. 2011	Benin	Implementation Science	Observation of antenatal consultations and patient exit interviews	5	4	5	5	5
Kelly et al. 2001	Kenya	American Journal of Public Health	Prospective cohort study over two years	5	5	5	5	5
Matthews et al. 1991	South Africa	South African Medical Journal	Cross-sectional descriptive community study over 2 weeks	5	5	5	5	5
Peltzer et al. 2012	South Africa	SAHARA-J: Journal of Social Aspects of HIV/AIDS	Two-arm randomized controlled trial	5	5	4	5	5
Ratsimbasoa et al. 2012	Madagascar	Malaria Journal	Non-randomised comparative study	4	4	3	3	4
Rennert & Koop 2009	Honduras	International Family Medicine	Prospective cohort study over 15 months	4	2	4	4	4
Rowe et al. 2007	Kenya	Transactions of the Royal Society of Tropical Medicine and Hygiene	Prospective cohort study over two months	5	5	5	5	5
Scott & Shanker 2010	India	AIDS Care: Psychological and Socio-medical Aspects of AIDS/HIV	Qualitative interviews, observations, and focus groups over 5 weeks	5	3	5	5	5
Soares et al. 2013	Brasil	International Journal of Tuberculosis and Lung Disease	Pre- and post test of 3 year intervention	3	4	3	3	3
Teela et al. 2009	Burma	Social Science & Medicine	Qualitative interviews, focus groups and case studies over two months	5	3	5	5	5
Torpey et al. 2008	Zambia	PLOS One	Cross-sectional descriptive community study with qualitative interviews and focus groups	5	5	5	5	5
Tulchinsky et al. 1997	West Bank	Journal of Public Health Management Practice	Pre- and post test of five year intervention	4	4	4	4	4
White & Speizer 2007	Zambia	BMC Health Services Research	Prospective cohort study	4	4	4	4	4
Wilkinson & Davies 1997	South Africa	Tropical Medicine and International Health	Prospective cohort study	5	5	4	5	5
Yansaneh et al. 2014	Sierra Leone	Tropical Medicine and International Health	Pre- and post test of 2 year intervention	3	4	3	3	3

### Observed measures and indicators of community integration

Table 2 provides an overview of the various measures of inclusion of information about integration of the community in the program, based on the four areas of interest (see method), split by quantitative (covariate) and qualitative measures (descriptions). The table also summarizes the relationship between these indicators and program effectiveness, measures of attrition, and program sustainability (see 3.3).

**Table 2:** Analysis of relationship between inclusion of community and impact indicators.

	Assessment of measured community integration variables	Assessment of qualitative community integration indicators				CLHW program outcomes		
	Quantitative measures of community integration included in analysis as covariate	Com-munity based program planning	Com-munity histories of recruits	Com-munity input in training	Com-munity engagement during implementation	Indicated effectiveness CLHW intervention	Indicated level of attrition CLHW	Indicated level of sustainability CLHW intervention
Adam et al. 2014	None	+/-		+	-	Effective. For women exposed to the health messages from CLHWs mean knowledge scores were higher (Eburru 32.3 vs 29.2, Kinale 21.8 vs 20.7, Nyakio 26.6 vs 23.8), more women delivered under skilled attendance, and percentage of facility deliveries increased (Eburru 46% vs 19%; Kinale 94% vs 73%: and Nyakio 80% vs 78%).	20%	A follow up at 1.5 years showed 80% community health workers still active. No other information.
Agboatwalla & Akram 1995	None	+/-	-	-	-	Effective. CLHW intervention group had better hygienic practices, more knowledge about ORS, diet, and vaccinations.	No data	Authors claim program became self-sustaining because of the integration of social (e.g. literacy) and health activities. They mention a rotating community fund to support CLHWs in literacy, sanitation and health activities. However, period of observation is for one year, and not measured beyond this point.
Baqui et al. 2009	None	-	+/-	+/-	+/-	Effective. Community health workers correctly classified very severe disease in newborns (91%) and almost all signs and symptoms (more than 60%).	50%	No longitudinal data available; comparison of physician assessment with CLHW assessment. High level of attrition in program is not further discussed.
Chang et al. 2009	None	+	-	-	+	Effective. However, study does not distinguish between roles of peer health workers and nurse clinicians in assessment of effectiveness.	No data	Program was effective over at least 2 years. Anecdotal evidence suggests strong religious roots and extensive existing community relationships helped increase effectiveness and sustainability of AIDS care program.
Chatterjee, et al. 2014	None	-	-	-	+/-	Modestly effective. Benefits most evident in the reduction of disabilities associated with schizophrenia and promotion of adherence to prescribed drugs. No difference in stigma or caregivers' understanding and knowledge of schizophrenia. Note this is a heavily supervised CHLW intervention, with high staff supervision costs.	No data	Observation over a one year period with no further detail on sustainability.
Colvin et al. 2003	None	+/-	+/-	-	-	Effective. Outcomes for DOT provided by traditional healers similar to other treatment supporters (treatment completion rate 88% vs. 75%, death rate 6% vs. 13%, default 6% vs. 11%, transfer 0% vs. 1%).	No data	No specifics. However, sustainability may have been jeopardized by noted levels of distrust between some traditional healers and medically trained treatment supporters.
Debpuur et al. 2002	None	+	-	+/-	-	Effective. The CLHW intervention reduced fertility in all treatment cells, but most prominently in areas where nurse outreach activities combined with community promotion (a 15% fertility decline relative to comparison communities).	No data	The authors note that longer-term observation is needed to rule out contextual causal influences as they believe minor and temporary lapses in program intensity can lead to widespread discontinuation of contraceptive use. No data available otherwise.
Dick et al. 1996	None	-	-	-	-	Partly effective. The CLHW intervention did not improve adult adherence to anti-tuberculosis treatment, but did for children. The latter is said to be the result of informal arrangements with community-based child-minders. The CLHW supervision option with staff achieved better adherence results for pre-school children.	No data	No specifics. The program was observed over 6 months.
Douthwaite & Ward 2005	None	-	-	-	-	Effective. The CLHW intervention increased likelihood of using modern reversible methods (OR ¼ 1.50, 95% CI ¼ 1.04–2.16, p ¼ 0.031). Authors note that restrictions in female mobility and the high value of modesty in this culture makes doorstep services through community-based female workers very effective.	No data	No specifics. Authors do note low prioritization of government-sponsored community and family planning services.
Dudley et al. 2003	None	-	-	-	+/-	Effective. The CLHW intervention (in the form of community supervision) achieved better outcomes than clinic-based intervention for both new smear-positive patients (cure rate: 72% vs. 46%; interruption rate 13% vs. 25%) and retreatment patients (cure rate: 63% vs. 35%; interruption rate 18% vs. 30%).	No data	Sustainable. While the data review treatment outcomes for a 2-year period, community-based TB care in the intervention site had been sustained over a 6-year period. It is noted that the numbers of treatment supporters and patients in community-based care increased over this time period, while performance of community-based TB care was maintained.
Frazão & Marques 2009	None	-	-	-	+/-	Effective. The CLHW intervention has positive outcomes. Authors relate this mostly to the role of social interactions helping to promote health practices, either during health consultations or by information exchange between mothers and women at family and community levels. There is little information about the content of the intervention.	13%	No specifics.
Gazi et al. 2005	None	-	-	-	+/-	Effective. The CLHW interventions were effective as service providers and promoters of health services in different types of urban setting. The authors note differences in impact across intervention sites.	53%	The authors suggest that retention may depend on the quality of supervision. They recognized that, as a result of the activities of depot-holders, the number of users had increased in the intervention year.
Hadi et al. 2003	None	-	+/-	-	+/-	Partly effective. The CLHW intervention is shown to be useful for identification and diagnosis of ARIs at grass roots, aggregate level, but not in very severe and severe cases. Authors note that the diagnosis and treatment were significantly more accurate among those health volunteers who had basic training and were routinely supervised.	“High”	Authors describe difficulties in providing basic training to a significant proportion (43%) of health volunteers, and a high need for intensive monitoring and close supervision of the program, and suggest that this is difficult to institutionalize in the longer term. They note the importance of including other health programs in the community.
Harvey et al. 2008	"Years as community health worker"	-	-	+	+/-	Effective. The CLHW intervention is an effective alternative for malaria case management in areas with limited microscopy, clinical personnel or facilities. The variable "years as community health worker" did not significantly affect overall performance.	No data	No specifics. No long-term data were collected. Authors note that while some amount of training seems critical for ensuring adequate performance, lengthy training programs can strain scarce health system resources both human and financial.
Jackson et a. 2013	None	-	-	-	-	Effective. The CLHWs are capable of safely conducting high-quality rapid HIV tests and interpreting the results. Home-based lay counselors achieved better results than clinic-based studies with professional nurses. Authors note that this may be due to the fact that lay counselors had extensive training and practical clinic-based experience prior to moving to the field.	No data	No specifics. The program was observed between September 2009 and January 2011.
Jacob et al. 2007	None	-	+/-	-	-	Ineffective. The CLHW intervention (training) produced very modest results. Authors argue that disorders with low prevalence cannot be diagnosed accurately at the community level unless a clinical referral system is in place which screens and confirms the diagnosis at multiple points.	No data	No specifics.
Jafar et al. 2010	None	-	-	-	-	Effective. The CLHW intervention ameliorated the usual increase in blood pressure with age in children and young adults. The authors note the conservative cultural context for this intervention, including restrictions in movement of women.	No data	The study duration was short, and authors note that it's therefore impossible to tell the extent to which changes in blood pressure can be sustained; nor can they speculate on the post-trial impact of the intervention, which has been variable in other trials.
Jennings et al. 2011	None	-	+/-	+/-	-	Effective. The CLHW had higher mean scores for general prenatal care and communication techniques, without significant increases in duration of antenatal consultations, as compared to nurse-midwives. The authors attribute this success to motivation, compliance to job aid instructions and interpersonal skills.	No data	No specifics. This is a short-term intervention training with no follow-up.
Kelly et al. 2001	None	-	+/-	-	-	Ineffective. The CLHW intervention showed deficiencies in the management of sick children, although care was not consistently poor.	No data	No specifics. The study observed the intervention over several years.
Matthews et al. 1991	None	-	-	-	-	Ineffective. The CLHW intervention does not increase knowledge of ORS in the community, even though over 80% of all respondents said they had previously known about or consulted the CLHW.	No data	No specifics. A cross-sectional descriptive community survey was conducted over 2 weeks.
Peltzer et al. 2012	None	-	-	-	-	Ineffective. Significant intervention effect between conditions was found.	No data	No specifics. A follow-up was done after 3 months. The authors note that other trials in Africa show that improved adherence might not persist over time.
Ratsimbasoa et al. 2012	None	+/-	+/-	-	+	Effective. The CLHW intervention shows high concordance between easy-to-use diagnostic tools at community level and microscopy.	No data	No specifics. Program was studied over a 24-month period and persisted during this period with the support of village level compensation.
Rennert & Koop 2009	Not a statistical methodology.	+	+/-	+	+	Effective. Three-monthly review and refresher sessions improved case management of respiratory tract disease, gastrointestinal infections, and skin infections.	No data	The program was observed 15 months after the intervention and compared with six months before. They note that its success can only be maintained with ongoing supervision, in-service training, and guidance.
Rowe et al. 2007	Multivariate model, including community women’s influence in CLHW selection and perceived benefits from the community.	-	+	-	-	Ineffective. The CLHW intervention showed no improvement of treatment-specific guideline adherence. Authors note that "non-intervention-related factors" were influential, including consultations performed by CLHWs. CLHWs thought they received benefits while working as a CLHW, including money, respect, happiness, gifts or help with chores as appreciation for duties.	No data	No specifics. Authors argue that results indicate that refresher trainings and supervision were ineffective and that this may hinder long-term sustainability.
Scott & Shanker 2010	Not a statistical methodology.	+	+	+	+	Effective. The CLHW intervention shows that immunization rates rose from 45% in 2002/2004 to 63% in 2007/2008, and antenatal checkups from 21 to 34%. CLHWs are reported to note enjoyment interacting with community members, while the position increased their social status.	No data	No specifics. Authors argue however that the sustainability of the program is limited as a result of remuneration structures, lack of institutional support, hierarchy in the health system, and a lack of community participation.
Soares et al. 2013	None	-	-	-	-	Effective. The CLHW intervention caused a rate decline by an average of 39 cases per 100000 population per 6 months. They note that this may have been due to secular trends already in place at the start of the intervention.	“minimize” (through stable employment contracts)	Authors note that drug and gang violence driven by external factors jeopardized the sustainability of this program.
Teela et al. 2009	Not a statistical methodology.	+	-	+/-	+	Partly effective. The CLHW intervention is effective for some emergency obstetric care services in community- or home-based settings. Authors note that delays in care seeking can be overcome with a strong focus on community trust and local ownership in the context of the militarily insecure environment.	24%	The intervention aims to deliver essential health services in these vulnerable communities while the military regime actively works to prevent services, targeting health care workers associated with ethnic groups. The negative security and logistical factors (distance, topography, weather) are obstacles to reaching the program goal, which therefore relies more on local community ownership. Authors argue for attention to social norms of care-seeking, gender, power, and traditional practices to make program more sustainable.
Torpey et al. 2008	None	-	-	-	+/-	Effective. The CLHW intervention reduced waiting times for adherence counseling and loss to follow-up rates of new clients declined from 15% to 0%.	9%	Authors note the significance of relatively low training cost for sustainability. The average cost of training one CLHW was approximately $320.
Tulchinsky et al. 1997	None	-	+/-	-	+/-	Effective. The CLHW intervention achieved high levels of access, participation, coverage, and utilization of preventive health care for prenatal, perinatal, and child care. This was achieved with high community support for relatively low cost.	10%	The program had been ongoing from 1987–1992 during difficult circumstances. Since initial UNICEF startup and despite the Intifada, the program was scaled up through various government institutions, including the Palestinian Authority. The authors suggest that this is testimony of strong community support. However, retention also appears to be maintained by high stipends for the CLHWs.
White & Speizer 2007	None	-	-	-	-	Effective. The CLHW intervention increased the likelihood of modern contraceptive use among rural women, while CLHWs were better able to maintain high treatment completion rates than health workers. This was achieved without exposing patients supervised by non-health workers to any excess risk of death.	No data	No specifics.
Wilkinson & Davies 1997	None	-	-	-	-	Effective. The intervention showed no difference in mortality between patients supervised by health workers (4%), community health workers (6%) or voluntary lay persons (5%). Further, the latter two groups were better able to maintain higher treatment completion rates than health workers.	No data	No specifics. The analysis is based on data for 1991 that refers to the period June to December.
Yansaneh et al. 2014	None	-	-	-	-	Not effective. The CLHW intervention did not appear to affect care-seeking from an appropriate provider, which increased in both study groups. Deployment of community health volunteers was associated with a reduced treatment burden at facilities and less reliance on traditional treatments.	No data	No specifics. Program was measured over two years.

As can be seen in Table 2, only two of the selected set of 32 studies from low- or middle-income countries included some measure of community integration as covariate in evaluation of effectiveness, attrition, or general sustainability. Harvey et al. 2008, in a study reviewing effectiveness of rapid diagnostic malaria tests, included the measure "years as community health worker", which proved to have no significant influence [37]. Rowe et al. [38] studied the effect of multiple interventions on CLHW adherence to clinical guidelines in Kenyan Siaya district, and included the variable “community women’s influence in CLHW selection” in a multivariate model with 12 covariates (of 75 examined). These authors also found no evidence that the involvement of community women in the CLHW selection process was associated in any significant way with overall or treatment-specific guideline adherence. While these initial results suggest that community integration may have no influence, the main result to be noted is the very lack of quantitative information on this issue within the other set of studies, excluding three qualitative oriented assessments [31,39,40].

Table 2 further shows that the lack of quantitative measures is echoed by a lack of information on qualitatively described indicators of community integration. It can be seen that most of the studies include very little description of community-based planning, recruitment through community-based resources, community input in training, or community engagement during implementation. Only ten studies provide a little detail on some of these topics [31,37–45] but none of the community integration indicators is comprehensively described by any of the studies selected. Below, we report for each sub-question what the studies did report.

### Community-based program planning and initiative

#### No data on community planning or initiatives at all

Twenty-one of the 25 studies did not make any reference to the community as driving or motivating the CLHW program. Twenty-two articles provide no information on the issue of community participation in program planning. In some cases this seems to be because partnerships at national or global levels drive the study initiative, and in others—such as the Pakistani “Lady health workers program”—despite emphasis on the notion that the program is decentralized (see also a related review article detailing the same program). In these articles there is no mention that these programs may have emerged from the communities themselves, or with community input. This includes six studies of CLHW programs that were devolved to local NGOs. A few studies test effectiveness of specific techniques or instruments (e.g. rapid diagnostic tests) and are typically initiated by research-oriented partnerships without consideration of community inputs or initiatives [37,45–48] (with one exception: the evaluation of a CLHW programme in Siaya district, Kenya by Rowe et al., who assessed the influence of health committees, soliciting the opinions of women in villages on guideline adherence [38]).

#### Too few details to be useful

Some studies mention the role of the community more explicitly, but provide too little detail to be useful for further analysis. Matthews et al. note the use of a participatory evaluation method in South Africa that motivated CLHWs to participate in the design of the evaluation of their work [49]. Gazi et al. note that in order to perform their tasks effectively, depot-holders (described as women from the community who promote good health practice and use of clinics and who keep a stock of contraceptives and oral rehydration salts) require the support of their families and of the community in general, and they report that most (over 80%) receive such support [43]. However, although they report that in the preliminary stages the NGOs informed local landlords, politicians, club members and local leaders about their activities to overcome problems with local gangsters, they do not say how this impacted the program. Colvin et al. report on the contribution of traditional healers to a rural tuberculosis control program in Hlabisa South Africa [50]. Although initiated from the outside, this pilot program builds explicitly on the possibility of using an indigenous network of traditional healers, offering the option of traditional healers in one sub-district for directly observed treatment (DOT) supervision. However, the article does not report that traditional healers had any influence on how this program was organized; they were “selected and trained” without much detail on how well the traditional healers themselves were integrated in their respective communities.

#### Community input acknowledged and (to some extent) described

Five studies stand out as acknowledging, in greater detail, that the programs in question were developed as part of community initiatives [31,39–42]. Rennert describes a program in Honduras, which was initiated through a privately funded U.S. hospital-based group—*The Brigade*—and is, as such, a top-down initiative [39]. However, the small support structure of this internationally driven initiative became, through necessity, dependent on local organization, forcing community involvement during the setting up of the program. The authors describe how a community health committee was required by the project in both of the target communities, to oversee them locally, with CLHWs in leadership roles. In each community, the community health committees were given autonomy in the selection process. Finally, Debpuur et al. report on the Navrongo Project’s impact on contraceptive use and knowledge, fertility and reproductive preferences [42]. Funded through the Ghana Ministry of Health to address the crisis in rural human resources for health, the study uses general demographic survey data to compare the impact of a traditional, indigenous social cooperation, termed the *zurugelu* approach, with the mobilization of support for community health planning using clinic-based, community health nurses and community health aides. The authors note that the *zurugelu* approach involves health care action committees that include elders, traditional peer networks, and linkages between supervision and traditional self-help schemes. The health aides, or *yezura zenna*, are chosen by the community.

### Histories of community recruits

#### Little information on who the CLHW actually were

Who are the community health workers in the studies? In about half of the studies testing for the impact of CLHW programs, we find little information on who the CLHW actually were, including their engagement with previous health roles and their ties to the communities. Fifteen studies provide little to no information about where CLHWs were recruited from or where they had been before their recruitment [37,42,43,45–49,51–58]. Where mentions are made, this is restricted to comments such as: “girls from the community having studied up to secondary school” [51]. Yansaneh et al. [59] and Kelly et al. [48] note in passing that CLHW volunteers were “selected by their communities”, but provide no further detail on how. Harvey et al. explain that most of the recruited CLHWs had preexisting experience with malaria treatment, but say nothing in the study about the background and previous community roles of the CLHWs other than a locality criterion: “All participating CLHWs lived in Chongwe or Chibombo District” [37]. This lack of information also characterizes the study by Debpuur et al. using the *zurugelu* approach [42]. Although the program includes indigenous networks, the article only notes that the CLHWs were recruited “using the traditional community system” and are called "*yezura zenna*".

#### Brief demographic descriptions

Five papers note that CLHW were recruited in new roles and provide brief demographic characteristics [22,60–62]. For example, Baqui et al. discuss how recruits are women with at least a 10th grade education, a mean age at time of recruitment of 23 years, and more than 60% of whom are single, divorced or separated [61]. Hadi et al. note that volunteers were selected from among the local area, most had five years of schooling, and were generally middle-aged and poor women [22]. The authors argue that because the volunteers were a homogeneous group of less educated, married women, they did not expect age variation to have a bearing on performance. In this set of studies, it seems that the two defining characteristics consistently mentioned are place-based—the recruits are from the community—and demographic. The study by Colvin et al implicitly provides information about the history of the recruits, as they were all traditional healers, although the authors do not go to any length explaining anything else about these people’s community histories [50].

#### Acknowledgment of importance of previous experience, but no testing

However, four studies do add information about community selection processes and inputs, and even to some extent about whether recruits have worked in community health roles before [38–40,50]. Teela et al. describe how all CLHWs had completed a minimum of 4 months training prior to the program, while making a commitment to work three years in their communities [40]. The CLHWs’ experience varied from reproductive to primary health care and had in all cases been at least two years. But none of these differences were tested. Adam et al. and Rennert et al. describe how the CLHWs recruited were respectively farmers [44], or either nuns or farmers, brought forward by a local committee organizing the program [39]. Although not much detail is given about the community relationship of the recruits, this is more detail than that provided in other studies. Another small group of studies focuses specifically on peer health workers, or people with previous experience of a specific disease as CLHW [41,63,64]. Here, previous experience is explicitly acknowledged, yet like most studies, very little additional information is available on these recruits and on their relationship with the community. In general, all of these studies restrict themselves to identification of the importance of recruitment in collaboration with communities, and acknowledgment that previous experience in health is desired, but with no testing of its impact on effectiveness. “Previous experience” remains largely unqualified but appears ideally to be clinical.

#### Previous experience focused on, with limited testing

Two studies are exceptions to the general pattern [31,38]. Scott et al. focus specifically on community relationships, also using a qualitative methodology, including focus groups, observation and interviews. They note how policies in India mandate CLHW recruitment through community mobilization processes and community-based accountability, yet the CLHWs were often selected without community consultation and are seen as entirely accountable to the primary health care unit, where they also received their remuneration [31]. Another exception is, once more, the evaluation study by Rowe et al. in Siaya district, Kenya, which focuses particularly on commonly made errors in managing childhood illness. The authors note that some villages established health committees to select CLHWs, while obtaining the opinions of women in the village [38]. The authors explain that they requested women’s opinions because most of the patients’ caretakers, with whom CLHWs interact, are mothers. They argue that women would contribute ideas about the characteristics of CLHWs important for providing good care, although the authors do not explain what characteristics surfaced in these discussions. They do, however, test for the impact of this selection process empirically (see 3.2).

### Community input in training

#### Little to no indication of community input in training

Nearly all studies give very little indication, or none at all, of community input in the CLHW training process and curriculum. Jacob et al describe an assessment of diagnostic capacity for dementia that includes community participation [60]. They asked CLHWs to nominate people with dementia based on their knowledge of the local community, and asked them to obtain information from health workers and key informants living in the village in order to reach a conclusion on people who might be suffering from the condition. It is not clear how much this training included community relationships or input. Five studies explicitly describe a community relations element in CLHW training, designed to improve community engagement. This included mostly capacity building in interpersonal communication skills, behavioral change communication, community entry and diplomacy [40,42,61,65].

#### Input from the community taken into account qualitatively

Only three studies took into account input from the community in training itself. Rennert et al. [39] mention that the Brigade developed a week-long training course specific to community needs, including a needs-based assessment for curriculum design, but no further detail is given. Adam et al. describe a community-based participatory process that was used to refine the spacing of the training schedule to accommodate the needs of the small-scale farmers volunteering to be CLHWs, and of their supportive community [44]. Finally, as mentioned above, there is the qualitative evaluation study of the Indian ASHA programme by Scott et al. [31]. Here, specific focus on community relationships illustrates the *lack* of community input, despite government policies mandating otherwise, eventually leading to mistrust between villagers and CLHWs.

### Community engagement during implementation

#### Little to no information about ongoing relationships and connections

Nearly all studies provide little to no information about the extent to which the CLHW program forged ongoing relationships and connections to community processes and dynamics to sustain motivation or create opportunities for input on program implementation by the community. Relationships are described in slightly more detail in eight studies [22,37,43,45,52,53,61,62,64]. For example, Gazi et al. dedicate a special section in their article to the interaction between depot holders and the community. They note that depot holders were generally valued for being community members, but leave out details on how the program intentionally engaged with the community. Ratimbasoa et al. note how in exchange for their participation, CLHWs received an annual allocation of rice as compensation for their work and help from the villagers to maintain their fields [45]. Overall, most of these studies lack detail on the way engagement proceeded, or how it contributed to decisions made during program implementation.

#### Structural constraints (e.g. distance, security) motivating engagement

Three studies appear to be closely related due to constraints in the program environment [39,40,66]. The qualitative study by Rennert et al. has already been described. Here, program health committees were set up to implement the US-funded initiative in two distant communities without any major national organization overseeing the work. The committees included mayors, a member from a local water board, the director of the local orphanage, two community elders, the principal and teachers from local schools, and a local church leader. Because the Brigade chose not to intervene in the health worker selection process beyond the basic criteria outlined above, the health committees developed autonomy during the program, although few details are given as to what this meant. Similarly, distance plays a role in promoting community engagement in the program studied by Teela et al., who document community-based delivery of maternal care in conflict-affected areas of eastern Burma [40]. The multi-ethnic collaboration of local community-based maternal and child health care organizations seeks explicitly to build community trust and confidence because of the special challenges which the negative security environment poses to reaching programmatic goals.

#### Inherent programmatic focus on community engagement

Only two programs studied appear to be motivated to develop community engagement to achieve programmatic gains. Chang et al. [41] detail a community-based program, including peer health educators (people living with HIV) that comprise about two thirds of the project staff. As a church initiative, the program involved extensive pre-existing community relationships which, according to the authors, helped to promote buy-in, follow-up, adherence, and dissemination of HIV care and prevention knowledge within the community. Agboatwalla & Akram report community support in the case of the Pakistani Health Education and Literacy Project (HELP) managed by a local NGO in the form of maintenance of activities, such as paying for CLHW services, and by monitoring of the project with little top-down supervision [51].

Of course mentioning community engagement does not in itself provide evidence that it is proceeding as envisaged. For example, Scott et al. observe that monthly Village Health Days or health planning sessions in which local people including CLHWs (ASHAs) are expected to participate in project planning do not occur as planned [31]. They document that *if* Village Health Days happen, these events feature nurses or local politicians lecturing the people about health issues and upcoming health events, such as immunization camps. The authors describe how the health system hierarchy limits opportunities for CLWHs to communicate up the chain of status and income.

## Synthesis of Findings

Based on the findings on each of the sub-questions, is it possible to identify evidence of a plausible relationship in low- or middle-income countries between the integration of community health resources on the one hand and CLHW effectiveness and sustainability on the other?

### Effectiveness

#### Integration of community health resources does not matter so much

With respect to effectiveness, most studies provide anecdotal evidence that the community relationship matters to program outcomes. Just three studies indicate indirectly that the quality of integration of community health resources in themselves do not matter much to outcomes [54,55,64]. Torpey et al. find that CLHWs are effective in adherence counseling despite only limited community interaction because of the mere practice of “following up”, regardless of the content of the interaction or the relationships built [64]. Similarly, White & Speizer find that mere CLHW outreach visits increase adherence to family planning methods [54]. Wilkingson & Davis find CLHW treatment to be effective without attention to community factors and attribute this success to decreased workload in health clinics [55].

#### Integration of community health resources matters

A few studies stand out for indicating more explicitly that attention to traditional roles and networks improves program effectiveness. Chang et al. find CLHWs effective in providing AIDS care [41]. While its authors do not distinguish between the roles of peer health workers and nurse clinicians in assessment of effectiveness, the study was initiated out of a church-based community network with community-based planning and finds very strong results of treatment adherence in a low-income setting, equal to those in high-income countries [41]. The qualitative assessment by Rennert et al. [39] concludes that the CLHW program was effective, with recruits coming from documented pre-existing roles within the community, including farming and nursing. The authors point out that while CLHWs donated their time to the project, the communities would support them in return during periods of need, such as harvesting. Similarly, Ratsimbasoa et al. [45] report on a successful program where in exchange for their participation, CLHWs received food staples (oil, rice), or help from villagers to maintain fields along with an annual allocation of rice. Teela et al. [40] report that the MOM program overcame delays in care seeking; the authors attribute this to strong focus on building community trust. Using qualitative data, the authors explicitly address topics such as the project introduction to the community, relationships with community members and leaders, and collaboration with health workers and traditional birth attendants, suggesting that these issues are central to the success of the intervention. The authors argue that a more refined framework for achieving improved access within a community-based program should consider factors such as social norms surrounding care-seeking, perceptions of the seriousness of obstetric emergencies, gender and power relations, household-decision-making, and traditional practices. Despite this, substantial obstacles and challenges remain in the context of a militarily insecure environment.

#### Combining community health resources with biomedical health outreach

In two other cases, the article by Debpuur et al. describing the *zurugela* approach [42] and the study by Colvin et al. [50], the contribution of traditional networks and roles is more specifically evaluated and even controlled for. Colvin et al. report that the outcomes of indigenous healers trained in the DOT program do not differ from other groups with respect to effectiveness, a positive result as expectations had been that they would be lower. They show that of 1,816 patients in Hlabisa District, there was no significant difference in treatment outcome comparing intervention and control areas (77% vs. 75%), or treatment completion (88% vs. 75%), while patients of traditional healers who had completed treatment revealed high levels of satisfaction with the care received. While Colvin et al. note distrust between some of the traditional healers and medically trained treatment staff, Debpuur et al. find that medical (nurse) and community-based indigenous health coalitions together provide the most effective results, a conclusion also reached by Dudley et al. [53]. Debpuur et al. find impact on fertility most pronounced when program outreach combines the involvement of nurses, traditional leaders and male volunteers. However, the authors note a lack of insight into the relative contribution of chiefs, elders, and social networks in reproductive health action mobilization, and suggest that more research is needed on this. While this is somewhat puzzling, considering that the study is focused on evaluating the effectiveness of an indigenous approach, this focus seems to have been more on male volunteers in family planning, and in this context, indigenous, traditional networks are defined as those of male dominance. Thus, the *zurugela* approach seems to try to use existing roles and community structures to strengthen community health by changing existing roles and structures

### Motivation & Attrition

#### Relevance of remuneration observed, but no testing for other motivators

Nine studies provide information about motivation, but do not connect this to retention or attrition [31,38,39,45,50,59,63,65,67]. With regard to attrition, the discourse in some studies suggests a particularly high relevance of remuneration [31,62,64,67]. Rowe et al. find that consultations performed by CLHWs who thought that they received four or more benefits had higher levels of overall patient adherence than consultations performed by CLHWs who thought that they received fewer than four benefits [38]. Dudley et al. note that although treatment supporters received R30 (US$4) per month per patient, the funding was limited and the sustainability of such projects was of serious concern to health managers and communities [53]. While strong views such as this are often expressed regarding the influence of remuneration in retention, this notion exists without insights into the complementary relevance of other non-monetary benefits and relationships. Three studies clearly note the importance of community recognition and social standing [31,50,59]. Aside from these factors, Jennings et al. explain significantly higher performance for communication on general prenatal care by lay nurse aides relative to midwives in a program in Benin [65]. They argue that this arises from recognition in the clinical field (by superiors), from the opportunity to be more involved in patient care, and from satisfaction with an expansion of professional competencies through capacity building.

#### Attrition data exist, but community relationships not investigated

Eight studies provide actual data on attrition [22,40,43,44,52,61,62,64], which ranges from 10% to 50% or “high”. High attrition—when discussed—is related to life events, a mismatch of expectations, or lack of supervision and monitoring. Baqui et al. find a 50% attrition rate but do not further discuss any of the causes for this, while Hadi finds “high” attrition in the ARI program and attributes this to a lack of adequate training, monitoring and supervision. Neither study describes community-based causes for attrition. In their study of depot-holders, Gazi et al. also find a 53% attrition rate, and attribute this to CLHWs finding other jobs, marriage, out-migration, sickness, greater volume of work than expected, and unsatisfactory performance in the eyes of the supervising institute. They note that many depot-holders considered the earnings insufficient and that some reported that they were embarrassed that their earnings were lower than those of housemaids. They detail additional problems in the community of Dhaka, which they attribute to alternative work opportunities and the general population’s greater involvement in cash economies. The authors do not further relate these identified causes for attrition to the noted importance of community dynamics, though they do describe the influence of "local conditions” and cite a number of factors influencing retention, including support from CLHWs’ families for their work (which seems high), a welcoming attitude towards CLHW activities, and the pride and social status expressed as a benefit by many CLHWs. At the low end of attrition, two studies ignore community relationships as possible causes for high retention rates. Frazao et al. find 13% attrition in a program promoting oral health [52], while Torpey et al describe a 9% attrition rate [64]. Both studies do note some community engagement during implementation, suggesting the importance of social interactions at family and community levels. Tulchinsky et al. find 10% attrition during ten years of operation which, as already noted, is related to a high stipend [62]. However, the authors also note that those who left did so for reasons “unrelated to the operations of the program” but do not specify what this means. Finally, the studies by Teela et al. [40] and Adams et al. [44] both find low attrition rates of around 20%, and both studies suggest a relatively high level of attention to community relationships during input and training/planning, and program implementation respectively. In both cases, causes for attrition are not specifically explained. Teela et al. do note negative security and logistical factors (distance, topography, weather) as severe program obstacles [40].

### Sustainability

#### No longitudinal data, or descriptive indications

Finally, with respect to sustainability, Debpuur et al. note how continuation of contraceptive use is vulnerable to even minor or temporary lapses in program intensity, as women readily abandon contraception if program support is disrupted [42]. From this perspective it is striking that at least sixteen studies provide no longitudinal data or descriptive indications of sustainability [37,45,46,48,49,52,54,55,58–61,63–65]. Soares et al. note that drug and gang violence driven by external factors jeopardized sustainability of the favela program [57]. Agboatwalle et al. claim that their program has become self-sustaining as a result of the integration of social (e.g. literacy) and health activities, indicating some relevance of community relationships at macro-level, yet they document only one year between pre- and post-intervention [51].

#### Importance of trust between community and health system

Some studies indicate the importance to sustainability of the relationship between supervisors and CLHWs, where CLHWs are positioned as members of communities with local connections. For example, Gazi et al. note that retention (and performance) of depot-holders depends on the extent to which supervisors value how much CLHWs know their locality and how they see this linked to increases in the number of users [43]. Colvin et al. note anecdotally how this very relationship, as it was to some extent characterized by distrust between traditional healers and medically trained professionals, could become an impediment to program sustainability [50]. A few studies stand out by virtue of the sustainability illustrated [31,40,41,44,53,62]. Chang et al. note that the alternative AIDS care program in Uganda was effective over at least a 2-year period, and attribute this sustainability explicitly to extensive community relationships, including community-based program planning. Teela et al. and Tulchinsky et al. make similar claims [40,62]. Scott & Shanker argue that it is precisely the lack of community participation, which, together with outcome-based remuneration structure, poor institutional support, and a rigid hierarchical structure of the health system, challenged the long-term sustainability of the ASHA program [31]. Considering the complex influences of, for example, political patronage in resource constrained settings, or patriarchal values influencing the care burden of the often female CLHW, it seems that accurate evaluations of CHLW programs in low-income countries still have a long way to go.

## Discussion

### Summary of evidence

Results show that only one of 32 studies from low- or middle income countries includes one statistical measure on indicators of community integration [38]. As a result of this lack of data it is difficult to derive an evidence-based conclusion for our propositions. Instead, our results indicate a larger problem, namely the complete *absence* of indicators measuring community relationships to the interventions or programs studied. What we find is a tendency for studies to refer to community or personal health worker factors as explanations for issues otherwise left unexplained (e.g. “informal arrangements” [68]). What is included are gender and peer-roles, complemented by limited demographic information about the recruits. The historicity of the health worker and the community s/he belongs to is absent in most studies reviewed. None of the studies discuss or test for the possibility that motivation emanates from the community. Only a few studies situate attrition and retention as an issue enabled by the community.

Results from other CLHW reviews support these findings, although none of these reviews focuses on community health resources specifically. In a U.S. based review, O’Brian finds that only 41% of reviewed studies included any discussion of the CLHW selection process [69]. He concludes that omitting CLHW selection or training procedures from the published literature neglects central information about the very intervention that is under scientific review and therefore hinders a complete understanding of the findings. Lassi et al., in a review of community-based intervention packages for improving maternal and neonatal outcomes, conclude that the most successful packages were those that involved family members through community support, advocacy groups and community mobilization & education strategies [70]. Yet, at the same time, they point out that most of the reviewed studies did not provide descriptions of the initial backgrounds of CLHWs deployed. In a well-known Cochrane review by Lewin et al. on lay health workers in primary and community health care, it is similarly noted that few studies documented the number of LHWs delivering care as well as selection or training, or levels of education (even though it appeared varied) [4]. Lewin concluded that few studies reported involving local people in the development of the interventions, the selection of LHWs, or the support of the LHW programs.

### Limitations

The review provided a synthesis of studies conducted in lower-income countries where health financing is generally low and the role of community health resources in the system more acute. This focus led to the exclusion of a body of knowledge developed in such countries as the United States, where CLHW initiatives have been much publicized. A further limitation has been the decision to focus on peer-reviewed articles only, as a result of which some innovative programmatic approaches are likely to have been excluded. Similarly, while there are many articles in the peer-reviewed scientific literature in which CLHWs play a critical role, some of these may have been excluded because the main purpose of this article is to assess the integration of community indicators in studies evaluating program effectiveness, rather than general descriptions highlighting how well a CLHW program functions. In addition, the databases searched were limited to the English language. While dominant in scientific publications, this may have biased results. Also, some recently published articles (2016) were not included in this review. Coding was done by only one of the authors, which may have also affected consistency and reliability of the findings. Finally, many of the articles generally focused on different topics, some of which included assessments of instruments by CLHWs as opposed to their performance in longer-term intervention programs, a few studies using a more qualitative assessment method. Despite this relative difference in focus and approach, the findings seem to be consistent overall. While the scope of the review’s findings is restricted by the limitations, the studies included in this review were generally of high quality and across a large span of time. This provided a strong basis for this review.

## Conclusion

We conclude that community relationships remain an under-reported resource in the evidence base of published, academic literature. Lacking such data, we are unable to assess whether the added value of programs building on strong community health resources makes a difference at all. Instead, we learn that in the body of peer-reviewed literature evaluating the effectiveness of CLHW programs, the false presumption has persisted that because CLHWs are installed and present, this automatically means that community relationship are also taken into account, dealt with, and their influence assumed to infiltrate up the health systems. As this is never made explicit, or questioned or problematized, the community essentially becomes a ‘black box” represented *by* the CLHW and varied in character only by paying some minimal attention to basic demographics, limited information about the clinical experience of the CLHW, and a non-historical interest in the peer role. Community participation and integration within program planning and implementation appears seen as complementary and supportive, but not central to the work of the community health worker. In this, we observe a fundamental misunderstanding of what a CLHW really is. Instead of a representative of a historical and place-specific community network, the CLHW is conceptualized as a labor unit, an interchangeable commodity. From this perspective, it is not surprise that solutions to improve CLHW programs continue to point towards the public health system, ignorant of the crucial need for strategic cooperation and shared learning [71].

## Supporting Information

S1 File Search Strategy. (DOCX)

S2 File Data extraction fields. (DOCX)

S3 File Protocol. (DOCX)
