# Supplementary material for: The Influence of Community Health Resources on Effectiveness and Sustainability of Community and Lay Health Worker Programs in Lower-Income Countries: A Systematic Review
Source: PLoS One. 2017 Jan 17;12(1):e0170217. doi: 10.1371/journal.pone.0170217 (PMC5240984; doi:10.1371/journal.pone.0170217)
Supplement: S1 File — (DOCX) [file pone.0170217.s001.docx]

**Appendix 1. Search strategy example**

**Medline**

*Ovid MEDLINE(R) In-Process & Other Non-Indexed Citations and Ovid MEDLINE(R)*

**#1 community health worker**

community health workers/ OR nurses' aides/ OR (((allied health* OR community health* OR community based health* OR health extension OR kinship OR lay health* OR lay nurse OR peer health* OR non-specialist health* OR village health* OR village malaria) ADJ2 (worker* OR activist* OR personnel* OR volunteer* OR aide*)) OR natural helper* OR barefoot doctor*).ti,ab,kf.

**Results: 9.142 (February 2, 2015)**

**#2 Low and middle income countries**

developing countries/ OR exp africa/ OR exp caribbean region/ OR exp central america/ OR "gulf of mexico"/ OR latin america/ OR mexico/ OR exp south america/ OR exp asia, central/ OR asia, southeastern/ OR exp asia, western/ OR china/ OR mongolia/ OR oceania/ OR caribbean region/ OR exp indian ocean islands/ OR indonesia/ OR pacific islands/ OR melanesia/ OR micronesia/ OR polynesia/ OR philippines/ OR west indies/ OR cuba/ OR dominica/ OR dominican republic/ OR grenada/ OR haiti/ OR jamaica/ OR "trinidad and tobago"/ OR (developing countr* OR afghanistan OR albania OR algeria OR angola OR argentina OR armenia OR azerb* OR bahrain OR bangladesh OR belarus OR belize OR benin OR bhutan OR bolivia OR bosnia OR brazil OR bulgaria OR burkina OR burma OR burundi OR cambodia OR cameroon OR africa* OR chad OR china OR colombia OR comoros OR congo OR cote d'ivoire OR ivory coast OR croatia OR cuba OR djibouti OR dominican republic OR ecuador OR egypt OR el salvador OR eritrea OR ethiopia OR fiji OR gabon OR gambia OR georgia OR ghana OR greece OR grenada OR guatemala OR guinea OR guyana OR haiti OR honduras OR india OR indonesia OR iran OR iraq OR jamaica OR jordan OR kazakhstan OR kenya OR kiribati OR korea OR kosovo OR kuwait OR kyrgyz* OR lao OR laos OR lebanon OR lesotho OR liberia OR libya OR macedonia OR madagascar OR malawi OR malaysia OR maldives OR mali OR marshall islands OR mauritania OR mexico OR micronesia OR moldova OR mongolia OR montenegro OR morocco OR mozambique OR myanmar OR namibia OR nauru OR nepal OR nicaragua OR niger OR nigeria OR oman OR pakistan OR palest* OR palau OR panama OR paraguay OR peru OR philippines OR romania OR russia* OR rwanda OR samoa OR "sao tome and principe" OR saudi arabia OR senegal OR serbia OR seychelles OR sierra leone OR solomon islands OR somalia OR south sudan OR sri lanka OR sudan OR surinam* OR swaziland OR syria* OR tajikistan OR tanzania OR thailand OR timor OR togo OR tonga OR "trinidad and tobago" OR tunisia OR turkey OR turkmenistan OR tuvalu OR uganda OR ukraine OR united arab emirates OR uzbekistan OR vanuatu OR venezuela OR vietnam OR west bank OR gaza OR yemen OR zambia OR zimbabwe).ti,ab,hw,kf.

**Results: 1.321.755 (February 2, 2015)**

NB. The list of countries is derived from the Worldwide Governance Indicators (WGI) database from the World Bank (<http://info.worldbank.org/governance/wgi/index.aspx#home> )

**1 AND 2 3.842 (February 2, 2015)**

**Limit to 2012 - current 880 (February 2, 2015)**

**Web of Science**

**#1 community health worker**

TS=((("allied health*" OR "community health*" OR "community based health*" OR "health extension" OR "kinship" OR "lay health*" OR "lay nurse" OR "peer health*" OR "non-specialist health*" OR "village health*" OR "village malaria") NEAR/2 ("worker*" OR "activist*" OR "personnel*" OR "volunteer*" OR "aide*")) OR "natural helper*" OR "barefoot doctor*")

**Results: 2.623 (February 2, 2014)**

**#2 Low and middle income countries**

TS=("developing countr*" OR "afghanistan" OR "albania" OR "algeria" OR "angola" OR "argentina" OR "armenia" OR "azerb*" OR "bahrain" OR "bangladesh" OR "belarus" OR "belize" OR "benin" OR "bhutan" OR "bolivia" OR "bosnia" OR "brazil" OR "bulgaria" OR "burkina" OR "burma" OR "burundi" OR "cambodia" OR "cameroon" OR "africa*" OR "chad" OR "china" OR "colombia" OR "comoros" OR "congo" OR "cote d'ivoire" OR "ivory coast" OR "croatia" OR "cuba" OR "djibouti" OR "dominican republic" OR "ecuador" OR "egypt" OR "el salvador" OR "eritrea" OR "ethiopia" OR "fiji" OR "gabon" OR "gambia" OR "georgia" OR "ghana" OR "greece" OR "grenada" OR "guatemala" OR "guinea" OR "guyana" OR "haiti" OR "honduras" OR "india" OR "indonesia" OR "iran" OR "iraq" OR "jamaica" OR "jordan" OR "kazakhstan" OR "kenya" OR "kiribati" OR "korea" OR "kosovo" OR "kuwait" OR "kyrgyz*" OR "lao" OR "laos" OR "lebanon" OR "lesotho" OR "liberia" OR "libya" OR "macedonia" OR "madagascar" OR "malawi" OR "malaysia" OR "maldives" OR "mali" OR "marshall islands" OR "mauritania" OR "mexico" OR "micronesia" OR "moldova" OR "mongolia" OR "montenegro" OR "morocco" OR "mozambique" OR "myanmar" OR "namibia" OR "nauru" OR "nepal" OR "nicaragua" OR "niger" OR "nigeria" OR "oman" OR "pakistan" OR "palest*" OR "palau" OR "panama" OR "paraguay" OR "peru" OR "philippines" OR "romania" OR "russia*" OR "rwanda" OR "samoa" OR "sao tome and principe" OR "saudi arabia" OR "senegal" OR "serbia" OR "seychelles" OR "sierra leone" OR "solomon islands" OR "somalia" OR "south sudan" OR "sri lanka" OR "sudan" OR "surinam*" OR "swaziland" OR "syria*" OR "tajikistan" OR "tanzania" OR "thailand" OR "timor" OR "togo" OR "tonga" OR "trinidad and tobago" OR "tunisia" OR "turkey" OR "turkmenistan" OR "tuvalu" OR "uganda" OR "ukraine" OR "united arab emirates" OR "uzbekistan" OR "vanuatu" OR "venezuela" OR "vietnam" OR "west bank" OR "gaza" OR "yemen" OR "zambia" OR "zimbabwe")

**Results: 1.963.245 (February 2, 2014)**

**1 AND 2 1.620 (February 2, 2015)**

**Limit to 2012 - current 724 (February 2, 2015)**

**Cochrane Library**

**#1 community health worker**

(("allied health*":ti,ab,kw OR "community health*":ti,ab,kw OR "community based health*":ti,ab,kw OR "health extension":ti,ab,kw OR "kinship":ti,ab,kw OR "lay health*":ti,ab,kw OR "lay nurse":ti,ab,kw OR "peer health*":ti,ab,kw OR "non-specialist health*":ti,ab,kw OR "village health*":ti,ab,kw OR "village malaria":ti,ab,kw) AND ("worker*":ti,ab,kw OR "activist*":ti,ab,kw OR "personnel*":ti,ab,kw OR "volunteer*":ti,ab,kw OR "aide*":ti,ab,kw)) OR "natural helper*":ti,ab,kw OR "barefoot doctor*":ti,ab,kw

**Results: 899 (February 2, 2015)**

**#2 Low and middle income countries**

"developing countr*":ti,ab,kw OR "afghanistan":ti,ab,kw OR "albania":ti,ab,kw OR "algeria":ti,ab,kw OR "angola":ti,ab,kw OR "argentina":ti,ab,kw OR "armenia":ti,ab,kw OR "azerb*":ti,ab,kw OR "bahrain":ti,ab,kw OR "bangladesh":ti,ab,kw OR "belarus":ti,ab,kw OR "belize":ti,ab,kw OR "benin":ti,ab,kw OR "bhutan":ti,ab,kw OR "bolivia":ti,ab,kw OR "bosnia":ti,ab,kw OR "brazil":ti,ab,kw OR "bulgaria":ti,ab,kw OR "burkina":ti,ab,kw OR "burma":ti,ab,kw OR "burundi":ti,ab,kw OR "cambodia":ti,ab,kw OR "cameroon":ti,ab,kw OR "africa*":ti,ab,kw OR "chad":ti,ab,kw OR "china":ti,ab,kw OR "colombia":ti,ab,kw OR "comoros":ti,ab,kw OR "congo":ti,ab,kw OR "cote d'ivoire":ti,ab,kw OR "ivory coast":ti,ab,kw OR "croatia":ti,ab,kw OR "cuba":ti,ab,kw OR "djibouti":ti,ab,kw OR "dominican republic":ti,ab,kw OR "ecuador":ti,ab,kw OR "egypt":ti,ab,kw OR "el salvador":ti,ab,kw OR "eritrea":ti,ab,kw OR "ethiopia":ti,ab,kw OR "fiji":ti,ab,kw OR "gabon":ti,ab,kw OR "gambia":ti,ab,kw OR "georgia":ti,ab,kw OR "ghana":ti,ab,kw OR "greece":ti,ab,kw OR "grenada":ti,ab,kw OR "guatemala":ti,ab,kw OR "guinea":ti,ab,kw OR "guyana":ti,ab,kw OR "haiti":ti,ab,kw OR "honduras":ti,ab,kw OR "india":ti,ab,kw OR "indonesia":ti,ab,kw OR "iran":ti,ab,kw OR "iraq":ti,ab,kw OR "jamaica":ti,ab,kw OR "jordan":ti,ab,kw OR "kazakhstan":ti,ab,kw OR "kenya":ti,ab,kw OR "kiribati":ti,ab,kw OR "korea":ti,ab,kw OR "kosovo":ti,ab,kw OR "kuwait":ti,ab,kw OR "kyrgyz*":ti,ab,kw OR "lao":ti,ab,kw OR "laos":ti,ab,kw OR "lebanon":ti,ab,kw OR "lesotho":ti,ab,kw OR "liberia":ti,ab,kw OR "libya":ti,ab,kw OR "macedonia":ti,ab,kw OR "madagascar":ti,ab,kw OR "malawi":ti,ab,kw OR "malaysia":ti,ab,kw OR "maldives":ti,ab,kw OR "mali":ti,ab,kw OR "marshall islands":ti,ab,kw OR "mauritania":ti,ab,kw OR "mexico":ti,ab,kw OR "micronesia":ti,ab,kw OR "moldova":ti,ab,kw OR "mongolia":ti,ab,kw OR "montenegro":ti,ab,kw OR "morocco":ti,ab,kw OR "mozambique":ti,ab,kw OR "myanmar":ti,ab,kw OR "namibia":ti,ab,kw OR "nauru":ti,ab,kw OR "nepal":ti,ab,kw OR "nicaragua":ti,ab,kw OR "niger":ti,ab,kw OR "nigeria":ti,ab,kw OR "oman":ti,ab,kw OR "pakistan":ti,ab,kw OR "palest*":ti,ab,kw OR "palau":ti,ab,kw OR "panama":ti,ab,kw OR "paraguay":ti,ab,kw OR "peru":ti,ab,kw OR "philippines":ti,ab,kw OR "romania":ti,ab,kw OR "russia*":ti,ab,kw OR "rwanda":ti,ab,kw OR "samoa":ti,ab,kw OR "sao tome and principe":ti,ab,kw OR "saudi arabia":ti,ab,kw OR "senegal":ti,ab,kw OR "serbia":ti,ab,kw OR "seychelles":ti,ab,kw OR "sierra leone":ti,ab,kw OR "solomon islands":ti,ab,kw OR "somalia":ti,ab,kw OR "south sudan":ti,ab,kw OR "sri lanka":ti,ab,kw OR "sudan":ti,ab,kw OR "surinam*":ti,ab,kw OR "swaziland":ti,ab,kw OR "syria*":ti,ab,kw OR "tajikistan":ti,ab,kw OR "tanzania":ti,ab,kw OR "thailand":ti,ab,kw OR "timor":ti,ab,kw OR "togo":ti,ab,kw OR "tonga":ti,ab,kw OR "trinidad and tobago":ti,ab,kw OR "tunisia":ti,ab,kw OR "turkey":ti,ab,kw OR "turkmenistan":ti,ab,kw OR "tuvalu":ti,ab,kw OR "uganda":ti,ab,kw OR "ukraine":ti,ab,kw OR "united arab emirates":ti,ab,kw OR "uzbekistan":ti,ab,kw OR "vanuatu":ti,ab,kw OR "venezuela":ti,ab,kw OR "vietnam":ti,ab,kw OR "west bank":ti,ab,kw OR "gaza":ti,ab,kw OR "yemen":ti,ab,kw OR "zambia":ti,ab,kw OR "zimbabwe":ti,ab,kw

**Results: 36.606 (February 2, 2015)**

**1 AND 2 392 (February 2, 2015)**

**Limit to 2012 - current 121 (February 2, 2015)**

**Sociological Abstracts**

*Proquest*

**#1 community health worker**

ALL((("allied health*" OR "community health*" OR "community based health*" OR "health extension" OR "kinship" OR "lay health*" OR "lay nurse" OR "peer health*" OR "non-specialist health*" OR "village health*" OR "village malaria") NEAR/2 ("worker*" OR "activist*" OR "personnel*" OR "volunteer*" OR "aide*")) OR "natural helper*" OR "barefoot doctor*")

**Results: 190 (February 2, 2015)**

**Peer Reviewed 130 (February 2, 2015)**
