# Supplementary material for: The Influence of Community Health Resources on Effectiveness and Sustainability of Community and Lay Health Worker Programs in Lower-Income Countries: A Systematic Review
Source: PLoS One. 2017 Jan 17;12(1):e0170217. doi: 10.1371/journal.pone.0170217 (PMC5240984; doi:10.1371/journal.pone.0170217)
Supplement: S2 File — (DOCX) [file pone.0170217.s002.docx]

**Appendix II: Data extraction form**

| Date to be documented | Answer category |
| --- | --- |
| Reviewer | Name |
| Author article | Open |
| Year | Open |
| Title | Open |
| Periodical | Open |
| The paper evaluates the effectiveness of CLHWs in a health program | Yes/No |
| The paper is published in a peer reviewed journal | Yes/No |
| The paper uses a randomized, quasi-randomized clinical trial or before/after methodology to test or evaluate the effectiveness of CLHW programs or alternatively has a substantial qualitative component supporting a descriptive assessment | Yes/No |
| The paper studies a CLHW program located in low or middle income country or region within country. | Yes/No |
| Type of data | 1 = Qualitative interviews |
|  | 2= observations |
|  | 3 = focus groups |
|  | 4 = cross sectional |
|  | 5 = pre- and post test |
|  | 6 = prospective cohort study |
|  | 7 = randomised controlled trial |
|  | 8 = comparative study |
| Aim of study | Open |
| Geographic location of study | Open |
| Describe to what extent the program/study builds upon indigenous networks | Open |
| Describe to what extent the initiative appears bottom-up | Open |
| Who took the initiative for the CLHW program? | Open |
| Describe to what extent the study documents if CLHWs are recruited in new roles | Open |
| Describe to what extent the study documents if CLHWs recruited have preexisting roles in community health | Open |
| Describe to what extent the study documents if who was recruited as CLHW | Open |
| Describe to what extent the study mentions or integrates traditional or indigenous roles | Open |
| Describe to what extent the study documents what training the CLHWs underwent | Open |
| Describe to what extent the study documents the motivation of CHWs (whether they are motivated and how they are motivated - i.e. what mechanisms)? | Open |
| Describe to what extent the study documents retention issues | Open |
| What attrition data can be found? | Open |
| For what period of time has the program been observed to be succesful? | Open |
| Describe the successes claimed by the CLHW program | Open |
| Describe to what extent the study documents community input in how the program is run | Open |
| Describe to what extent the study documents alternative health resources used to make the program run successfully | Open |
| Describe to what extent the study documents how health information/messages are transmitted | Open |
| Additional remarks or notes | Open |
