# Supplementary material for: The Influence of Community Health Resources on Effectiveness and Sustainability of Community and Lay Health Worker Programs in Lower-Income Countries: A Systematic Review
Source: PLoS One. 2017 Jan 17;12(1):e0170217. doi: 10.1371/journal.pone.0170217 (PMC5240984; doi:10.1371/journal.pone.0170217)
Supplement: S3 File — (DOCX) [file pone.0170217.s003.docx]

**Review title**

The Influence of Community Health Resources on Effectiveness and Sustainability of Community and Lay Health Worker Programs: A Systematic Review

**Reviewers**

Daniel H. de Vries, Ph.D., University of Amsterdam, Department of Anthropology, email: [d.h.devries@uva.nl](mailto:d.h.devries@uva.nl)

Rob Hoogenboom, M.A., University of Amsterdam, Department of Anthropology

Robert Pool, Ph.D., University of Amsterdam, Department of Anthropology

**Review question/objective**

Are CLHW programs that are inclusive and sensitive to indigenous community roles, groups and networks during program design phases intrinsically more effective and sustainable than those built on new roles and groups that are developed and implemented from the outside? We seek to identify peer-reviewed studies that evaluate CLHW programs in low or middle income settings using either a randomized, quasi-randomized or before/after evaluative methodology. The population of interest in this study is the CLHW participating in health programs in low or middle income settings. The intervention pertains to the influence of indicators of community integration in programmatic outcomes. Based on fieldwork experience through a study on community health resources in Uganda (CoHeRe), we specifically chose to pay attention to four potential indicators of community integration:

1. Community based program planning: To what extent are the reviewed programs building upon pre-existing indigenous networks and social roles? Who took the initiative for the CHW program? And how much input was there from community in the planning of the program before its implementation?
2. To what extent were the recruited CHW already engaged in community health roles? Were the community health workers recruited to work in traditional or indigenous roles that have historical ties to the community, or where these new CHW roles?
3. After recruitment, to what extent did community members have input in the training curriculum, and was the training based on experience inside relevant communities?
4. To what extent did the CHW program forge ongoing relationships and connections to community processes and dynamics? What connections are made to motivation of CHWs and the integration of the program within the community?

**Background**

The coincidence of the halfway mark to the millennium development goals (MDGs) with the 30th anniversary of Alma-Ata stimulated discussion about the role of a revitalized primary health care in strengthening health systems [1,2]. One of the lasting impressions of these discussions is the difficulty of motivating community ownership and participation in health, including the successful expansion of community health workers. Explicitly addressed as one of Alma-Ata’s principles, the ability of communities to participate in health service delivery appears to have been one of the less achieved elements of the Alma-Ata philosophy. The effectiveness of community health worker programs has been considered “patchy”, with difficulties in scale-up, an observed lack of consistent supervision, weak linkages to existing health systems, and no sustained community financing [3-6]. Unfortunately, in the new United Nations Sustainable Development Goals, community participation does not surface as a central theme in any of the formulations, with the exception of the goal to ensure availability and sustainable management of water and sanitation (Goal 6./b) [7]. Yet, the increasing awareness of a global shortage of human resources for health [8,9] as well as the observed inequity in health systems [10,11] emphasizes the continued need to strengthen linkages to the community and reinstate community health workers [1,2].

To achieve effective and sustainable community participation, we propose that health service delivery systems should emerge from the way in which health is organized at the community level. Our hypothesis is that inclusion of local structures, networks and roles that do not necessarily have an explicit medical function into programmatic design increases the effectiveness and sustainability of community and lay health worker programs. The aim of this systematic review therefore is to assess what empirical evidence exists that may confirm this proposition. We define community health workers as a broad category of lay workers that have been identified as able to carry out functions related to healthcare delivery at the community level without a formal professional or paraprofessional certiﬁcate or tertiary education degree. In the literature and in practice this rising health cadre has been referred to in various terms, most commonly and historically as “community health worker”, but also as “peer health worker”, “non-professional health care worker” or “lay health worker”. In this paper we will refer to this cadre as community and lay health workers (CLHW)

The lay worker concept in health service delivery received much initial enthusiasm in the 1980s, but waned as scaling-up of local CLHW programs models appeared difficult [3]. One major issue that plagues community and lay health worker programs is high levels of attrition resulting from resignations, terminations, or relocations [2,4,12], leaving the few enthusiastic and reliable lay workers that remain to become overloaded with work [13-15]. Attrition rates of up to 30% over 9 months in Senegal and 50% over 2 years in Nigeria have been reported [16], while Olang'o et al. report an attrition rate of 33% among home based care community health workers in western Kenya [17]. Furthermore, although community-based lay health workers can be volunteers [18], in practice most are financially rewarded, though there are hardly any examples of sustained community financing [19]. High attrition rates reduce the stability of programs, increase training costs because of the need for continuous replacement, and makes programs difficult to manage [20-22]. Moreover, fee-for-service payments may encourage inappropriate treatment [2].

Despite the significant impact of community and lay health worker attrition on programmatic stability and effectiveness, a World Health Bulletin points to the paucity of data on this issue [23]. The authors argue that lay worker attrition has not been a measurement priority nor a research priority. We believe that this conclusion is remarkable if attrition is seen as a factor leading to a lack of continuity in the relationship between CLHWs and their community. The question emerges to what extent the quality of the relationship between the community and the CLHW is in fact essential for the community health worker programs to work effectively and sustainably. An initial reading of the literature suggests the community relationship is indeed of significant importance. In a global review, UNICEF notes that well-functioning CLHW programs are partly dependent on frequent interactions with community members [19]. While it is noted that supervision is often among the weakest links in many CLHW programs, it has also been found that supervision is mostly effective at small scales only because a signiﬁcant amount of supervision and oversight comes from the community itself [24]. There is evidence suggesting that acceptance, support, and respect from the community as well as from the formal health system is essential for CLHWs to be motivated and effective, and that CLHWs should be selected on the basis of their motivation to serve the community in which they work in [25]. Belonging to the community is crucial because, ultimately, the success of the CLHW program is measured at the community level [26,27]. Landon qualitatively documented an Alaskan CLHW program where “high retention communities” received more emergency, financial and material support as well as respect from the community and greater responsiveness from village councils [21]. Others note that wherever selection of a CLHW has not been carefully considered, this can lead to a lack of trust from the community and become a contributing factor to high turnover [17,26,28]. Also noted is the need to pay attention to the economic and cultural environment within which CLHWs operate [17], such as gender norms [29].

**Inclusion criteria**

1. The paper evaluates the effectiveness of CLHWs in a health program
2. The paper is published in a peer reviewed journal
3. The paper uses a randomized, quasi-randomized clinical trial or before/after methodology to test or evaluate the effectiveness of CLHW programs or alternatively has a substantial qualitative component supporting a descriptive assessment
4. The paper studies a CLHW program located in low or middle income country or region within country. We excluded high-income countries because of the higher likelihood that CLHWs are financially remunerated and worked in an environment with many more public extension services, making lessons learned less comparable.

**Search strategy**

The literature search will be conducted in two phases. A first search without time-limit will be done in using the Cochrane Library review abstracts, Academic search premier, Web of science, Science Direct, Google Scholar, PubMed, and Annual Reviews. A number of journals will be hand-searched, in particular the Journal of Human Resources for Health, and references of particularly relevant full-text articles were also searched. Using these results, a second more automated search will be conducted in to complement the first, using Medline, Web of Science, Cochrane Library and Sociological Abstracts. For the second search, the following English search strategy was eventually used: community health workers/ OR nurses' aides/ OR (((allied health* OR community health* OR community based health* OR health extension OR kinship OR lay health* OR lay nurse OR peer health* OR non-specialist health* OR village health* OR village malaria) ADJ2 (worker* OR activist* OR personnel* OR volunteer* OR aide*)) OR natural helper* OR barefoot doctor*). This search was reduced by a selection of low and middle income countries derived from the Worldwide Governance Indicators (WGI) database , and undoubled. Studies identified were included in a RefWorks bibliographic database and organized according to extent to which they fitted the categories of effectiveness studies, review articles, or other contextual or qualitative narratives. The software was used to identify duplicates.

**Assessment of methodological quality**

The two reviewers independently conducted a review of abstracts using the above mentioned search strategy. Both reviewer agreed upon the articles to be used for selection in the second round of searches. Any disagreements that arise between the reviewers will be resolved through discussion, or with the second author. To assess publication bias of selected publications we used a critical appraisal process that involves (i) filtering against minimum criteria, involving adequacy of reporting detail on the data sampling, collection and-analysis, (ii) technical rigor of the study elements indicating methodological soundness and (iii) paradigmatic sufficiency, referring to researchers’ responsiveness to data and theoretical consistency [35]. Each of the studies was reviewed by the first author for these qualities and a review sheet was created in excel. This process was verified by the second author. Any disagreements that arise between the authors will be resolved through discussion.

**Data collection**

Both authors agreed upon the data extraction form, which was created in excel and used to integrate information regarding the sub-questions. The standardized data extraction tool is shown in Appendix I. The data extracted will include specific details about the interventions, populations, study methods and outcomes of significance to the review question and specific objectives.

**Data synthesis**

Quantitative papers will, where possible be pooled in statistical meta-analysis. All results will be subject to double data entry. Effect sizes expressed as odds ratio (for categorical data) and weighted mean differences (for continuous data) and their 95% confidence intervals will be calculated for analysis #modify text as appropriate#. Heterogeneity will be assessed statistically using the standard Chi-square and also explored using subgroup analyses based on the different quantitative study designs included in this review. Where statistical pooling is not possible the findings will be presented in narrative form including tables and figures to aid in data presentation where appropriate.

Qualitative research findings will, where possible be pooled. This will involve the aggregation or synthesis of findings to generate a set of statements that represent that aggregation, through assembling the findings rated according to their quality, and categorizing these findings on the basis of similarity in meaning. These categories are then subjected to a meta-synthesis in order to produce a single comprehensive set of synthesized findings that can be used as a basis for evidence-based practice. Where textual pooling is not possible the findings will be presented in narrative form.

**Conflicts of interest**

No authors have any competing interests. An ethics statement was not required for this work. Time for this review was funded through a Netherlands Organisation for Scientific Research (NWO) Global Health Policy and Health Systems Research grant. The NWO did not play any role in the design, writing or decision to publish this manuscript.

**Acknowledgements**

The authors would like to acknowledge Ms. Janneke Staaks for her librarian support.

**References**

1. Travis P, Bennett S, Haines A, Pang T, Bhutta Z, Hyder AA, et al. Overcoming health-systems constraints to achieve the Millennium Development Goals. The Lancet. 2004;364(9437): 900-906.

2. Haines A, Sanders D, Lehmann U, Rowe AK, Lawn JE, Jan S, et al. Achieving child survival goals: potential contribution of community health workers. The Lancet. 2007;369: 2121-2131.

3. Walt G, Perera M, Heggenhougen K. Are large-scale volunteer community health worker programmes feasible? The case of Sri Lanka. Soc Sci Med. 1989;29: 599-608.

4. Lewin S, Munabi-Babigumira S, Glenton C, Daniels K, Bosch-Capblanch X, VanWyk BE, et al. Lay health workers in primary and community health care for maternal and child health and the management of infectious diseases. Cochrane Database Syst Rev. 2010;20051-108.

5. Glenton C, Lewin S, Scheel IB. Still too little qualitative research to shed light on results from reviews of effectiveness trials: A case study of a Cochrane review on the use of lay health workers. Implement Sci. 2011;6: 53.

6. Lawn JE, Rohde J, Rifkin S, Were M, Paul VK, Chopra M. Alma-Ata 30 years on: revolutionary, relevant, and time to revitalise. The Lancet. 2008;372: 917-27.

7. United Nations General Assembly. Transforming our world: the 2030 Agenda for Sustainable Development. Geneva: United Nations. 2015;A/RES/70/1: 70/1.

8. WHO. The world health report 2006: working together for health. Geneva: World Health Organization; 2006.

9. WHO. Human resources for health: overcoming the crisis. Sustainability, mobilisation and increased knowledge key to strengthened health work force. Joint Learning Initiative ed. Geneva: World Health Organization; 2004.

10. Gwatkin DR, Bhuiya A, Victora CG. Making health systems more equitable. The Lancet. 2004;364: 1273-1280.

11. Walley J, Lawn JE, Tinker A, de Francisco A, Chopra M, Rudan I, et al. Primary health care: making Alma-Ata a reality. The Lancet. 2008;372: 1001.

12. Morrow RH. Commentary: lay health workers in primary and community health care. Int J Epidemiol. 2005;34: 1252.

13. Pisani E, Schwartländer B, Cherney S, Winter A. Report on the global HIV/AIDS epidemic. Geneva: Joint United Nations Programme on HIV/AIDS; 2000.

14. Battacharyya K, Winch P, LeBan K, Tien M. Community health worker incentives and disincentive: how they affect motivation, retention and sustainability. Arlington: United States Agency for International Development; 2001.

15. Akintola O. What motivates people to volunteer? the case of volunteer AIDS caregivers in faith-based organizations in KwaZulu-Natal, South Africa. Health Policy Plan. 2011;26: 53-62.

16. Parlato M, Favin M. Primary health care. Progress and problems: an analysis of 52 AID assisted projects. Washington D C : American Public Health Association. 1982.

17. Olang'o CO, Nyamongo IK, Aagaard-Hansen J. Staff attrition among community health workers in home-based care programmes for people living with HIV and AIDS in western Kenya. Health Policy. 2010;97: 232-237.

18. Walt G. CHWs:Are national programmes in crisis? Health Policy Plan. 1988;3: 1.

19. UNICEF. What works for children in South Asia: Community Health Workers. Kathmandu: The United Nations Children’s Fund; 2004.

20. De Zoysa I, Cole King S. Remuneration of the community health worker: What are the options? World Health Forum. 1983;4(2): 125-130.

21. Landon B, Loudon J, Selle M, Doucette S. Factors influencing the retention and attrition of community health aides/practitioners in Alaska. J Rural Health. 2004;20: 221-230.

22. Hadi A. Management of acute respiratory infections by community health volunteers: experience of Bangladesh Rural Advancement Committee (BRAC). Bulletin of the World Health Organization. 2003;81: 183-189.

23. Nkonki L, Cliff J, Sanders D. Lay health worker attrition: important but often ignored. Bulletin of the World Health Organization. 2011;89: 919-923.

24. Hermann K, Van Damme W, Pariyo GW, Schouten E, Assefa Y, Cirera A, et al. Community health workers for ART in sub-Saharan Africa: learning from experience--capitalizing on new opportunities. Hum Resour Health. 2009;7: 31.

25. Jaskiewicz W, Tulenko K. Increasing community health worker productivity and effectiveness: a review of the influence of the work environment. Hum Resour Health. 2012;10: 38.

26. Prasad BM, Muraleedharan VR. Community health workers: a review of concepts, practice and policy concerns. London: International Consortium for Research on Equitable Health Systems; 2007.

27. May ML. Promotor(a)s, the organizations in which they work, and an emerging paradox: How organizational structure and scope impact promotor(a)s work. Health Policy. 2007;82: 153-166.

28. Lehmann U, Sanders D. Community health workers: What do we know about them?   The state of the evidence on programmes,  activities, costs and impact on health outcomes of using community health workers. Geneva: World Health Organization; 2007.

29. Kironde S, Klaasen S. What motivates lay volunteers in high burden but resource-limited tuberculosis control programmes? Perceptions from the Northern Cape province, South Africa. Int J Tuberc Lung Dis. 2002;6: 104-110.

**Appendix I: Data Extraction Tool**

| Date to be documented | Answer category |
| --- | --- |
| Reviewer | Name |
| Author article | Open |
| Year | Open |
| Title | Open |
| Periodical | Open |
| The paper evaluates the effectiveness of CLHWs in a health program | Yes/No |
| The paper is published in a peer reviewed journal | Yes/No |
| The paper uses a randomized, quasi-randomized clinical trial or before/after methodology to test or evaluate the effectiveness of CLHW programs or alternatively has a substantial qualitative component supporting a descriptive assessment | Yes/No |
| The paper studies a CLHW program located in low or middle income country or region within country. | Yes/No |
| Type of data |  |
| 1 = Qualitative interviews  2= observations  3 = focus groups  4 = cross sectional  5 = pre- and post test  6 = prospective cohort study  7 = randomized controlled trial  8 = comparative study | |
| Aim of study | Open |
| Geographic location of study | Open |
| Describe to what extent the program/study builds upon indigenous networks | Open |
| Describe to what extent the initiative appears bottom-up | Open |
| Who took the initiative for the CLHW program? | Open |
| Describe to what extent the study documents if CLHWs are recruited in new roles | Open |
| Describe to what extent the study documents if CLHWs recruited have preexisting roles in community health | Open |
| Describe to what extent the study documents if who was recruited as CLHW | Open |
| Describe to what extent the study mentions or integrates traditional or indigenous roles | Open |
| Describe to what extent the study documents what training the CLHWs underwent | Open |
| Describe to what extent the study documents the motivation of CHWs (whether they are motivated and how they are motivated - i.e. what mechanisms)? | Open |
| Describe to what extent the study documents retention issues | Open |
| What attrition data can be found? | Open |
| For what period of time has the program been observed to be succesful? | Open |
| Describe the successes claimed by the CLHW program | Open |
| Describe to what extent the study documents community input in how the program is run | Open |
| Describe to what extent the study documents alternative health resources used to make the program run successfully | Open |
| Describe to what extent the study documents how health information/messages are transmitted | Open |
| Additional remarks or notes | Open |
